# Discriminative validity of summarized hip and knee angular accelerations for lower extremity training load quantification in male soccer players during a standardised training drill

**DOI:** 10.1080/24733938.2023.2290083

**Published:** 2023-12-07

**Authors:** Bram J. C. Bastiaansen, Riemer J. K. Vegter, Erik Wilmes, Edwin Goedhart, Koen A. P.M. Lemmink, Michel S. Brink

**Affiliations:** a Center for Human Movement Sciences, University of Groningen, University Medical Center Groningen, Groningen, the Netherlands; b Amsterdam Movement Sciences, Department of Human Movement Sciences, Faculty of Behavioural and Movement Sciences, Vrije Universiteit Amsterdam, Amsterdam, The Netherlands; c FIFA Medical Centre of Excellence, Royal Netherlands Football Association, Zeist, The Netherlands

**Keywords:** Football, IMU, intermittent exercise, kinematics, validation study

## Abstract

This study assessed the discriminative validity of summarized hip and knee angular accelerations during a standardized training drill. Twenty-eight soccer players performed a standardized training drill that mimics game demands. Discriminative validity was examined by assessment of between-group differences of summarized preferred kicking leg hip and knee angular accelerations, and Playerload between national and regional soccer players for the full training drill, and parts based on locomotor intensity, or additional pass and jumping header activities. Furthermore, relationships were assessed between the summarized hip and knee angular accelerations and conventional load indicators derived from a local positioning measurement system, such as high-intensity running distance and Playerload. National players had higher summarized hip (Mean difference: 62.7 A.U. ES = 0.77, *p* = 0.049) and knee (Mean difference: 137.1 A.U. ES = 1.06, *p* = 0.008) angular accelerations. Significant interaction effects were observed during high-intensity running (Hip: 0.2 A.U./m, ES = 0.98, *p* = 0.005; Knee: 0.61 A.U./m, ES = 1.52, *p* < 0.001), and sprinting (Hip: 0.3 A.U./m, ES = 1.01, *p* < 0.02; Knee: 0.56 A.U./m, ES = 1.57, *p* < 0.001). Between-group differences were not present for additional passing or jumping header activities. Compared to summarized hip and knee angular accelerations, Playerload had less ability to discriminate between players and activities. Moreover, the lower extremity training load indicators were unrelated to conventional load indicators. Together these results confirm discriminative validity of summarized hip and knee angular acceleration training load indicators during a standardised training drill.

## PRACTICAL IMPLICATIONS

The results in this study substantiate the use and validity of summarized hip and knee angular accelerations as external training load indicators for the lower extremities in soccer. Based on the known-group difference technique, expected differences in summarized hip and knee angular accelerations between national and regional soccer players were observed during a standardized soccer training drill. These differences were present during high intensity running and sprint activities, but not for passing or jumping header activities. The limited ability of Playerload to discriminate between players and tasks, and insignificant relationships between measurement systems in this study demonstrate that summarized hip and knee angular accelerations can be used complementary to whole-body training load indicators for evaluating soccer training and match play. The presented methodology provides insights in the training load of individual limbs, which potentially can be used to assess the effectiveness of specific training drills to under- or overload specific limbs. Trainers, coaches, and sport practitioners can use this information to balance training load and recovery in soccer, aiming to improve performance and prevent overload.

## Introduction

Soccer players train to achieve physical requirements and improve physical performance of the game. For optimal performance, the frequency, duration, and intensity of exercise (i.e., training load) can be regulated that induce positive adaptations when sufficient recovery is provided (Smith 2003). Training load can be described in terms of player activities on the field (*i.e.*, external load), their response (*i.e.*, internal load) or from a physiological or biomechanical perspective (Vanrenterghem et al. 2017). By quantifying the training load with monitoring systems and providing feedback to athletes, coaches and other sports professionals, insights are given about the training process. This could help to evaluate whether the desired training outcomes were met and to intervene when necessary.

External load monitoring is frequently based on a combination of whole-body position registration systems and an integrated inertial sensor (Akenhead and Nassis 2016). This allows for derivation of total distance and distance covered between pre-defined speed and acceleration thresholds, or Playerload (Boyd et al. 2011). Such indicators provide useful insights in the locomotor demands of soccer training and match play. However, these methods do not fully capture the lower extremity load during soccer-specific activities with small locomotor displacements, such as passing, kicking or jumping (Wilmes et al. 2022). As these activities significantly stress the musculoskeletal tissues in the lower extremities (Naito et al. 2010), solely monitoring locomotor activities might result in an underestimation of training load that players endure. Since biomechanical load-adaptational pathways are different from physiological pathways (Vanrenterghem et al. 2017), the current inability to quantify lower extremity training loads limits adequate periodization and performance optimization strategies.

Current field-based methods are unable to quantify stresses and strains, or (repetition of) forces on musculoskeletal tissues (Verheul et al. 2020). However, it is possible to register lower extremity kinematics in the field with a validated inertial sensor setup (Wilmes et al. 2020; Bastiaansen et al. 2020), which makes it feasible to derive training load indicators specific for the lower extremities. Human movement is mainly driven by net joint torques originating from external forces (e.g. ground reaction forces), muscle contractions, ligaments, and other tissues crossing the joint (Zajac and Gordon 1989). This results in an angular acceleration across a joint that can be measured with the inertial sensor setup. Lab-studies showed that musculoskeletal tissues are significant contributors to net torques (Pandy and Zajac 1991; Naito et al. 2010; Huang et al. 2013), and thus joint angular accelerations might provide better insights in the lower extremity loads compared to whole-body load indicators.

Hip and Knee Load (Equation 2) were therefore introduced as training load indicators for the lower extremities (Bastiaansen et al. 2022). It must be recognized that the term ‘load’ does not refer to actual measured forces but refers to the construct to describe player activities in the field by quantification of angular accelerations (Impellizzeri et al. 2022). Bearing this in mind, we will proceed with summarized hip and knee angular accelerations throughout the manuscript for transparency and to avoid misinterpretation. Previous work showed that the summarized hip and knee angular accelerations were sensitive for increased movement intensity and additional kicking or jumping during soccer specific shuttle runs, with acceptable to good test-retest reliability (Wilmes et al. 2022). Discriminative validity between groups was assessed in another study, which reported higher summarized knee angular acceleration values for national players during jumping and kicking (Bastiaansen et al. 2023). Furthermore, exploration of relationships between measurement systems during a sprint and agility task revealed that summarized hip and knee angular accelerations were unrelated to load indicators derived from conventional methods, such as Playerload or high intensity running distance (Bastiaansen et al. 2022). These results demonstrate the added value of summarized hip and knee angular accelerations as training load indicators for the lower extremities measured in the field.

The above-mentioned studies were, however, performed under standardised situations. Furthermore, participants were instructed to perform kicking or jumping header tasks at maximal intensity to increase contrast between conditions. However, these tasks do not fully represent the movement patterns and technical requirements of real match play. For example, the effects of targeted passing behaviour with less emphasis on maximal kicking intensity remain relatively unexplored. To replicate these demands, conditions can be simulated during training, such as during small-sided games (Dellal et al. 2011). The effectiveness of small-sided games can, however, be limited by organisational factors, such as number of players or pitch dimensions, and thus not always elicit the desired training outcome. Alternatively, soccer-specific training drills were developed (Kelly et al. 2013), which include a controlled activity pattern replicating the movements and technical requirements of the game. During soccer-specific training drills, the validity of summarized hip and knee angular accelerations can be assessed under more valid ecological circumstances. Previous studies showed higher summarized angular accelerations with increased movement intensity during soccer-specific tasks (Lees et al. 2004; Nunome et al. 2006; Schache et al. 2011). Furthermore, national players cover more high-intensity distance, have better passing performance in small sided games (Dellal et al. 2011; Klingner et al. 2021) and achieve higher ball speeds (Kellis and Katis 2007), presumably because national players are better trained and in better physical condition compared to regional players. Since summarized hip and knee angular accelerations are sensitive to movement intensity and additional lower extremity load with jumping or kicking activities (Wilmes et al. 2022), mechanical stress on lower extremity tissues is assumed to be higher during training drills, especially at maximal intensity, for national players compared to regional players because national players are generally better trained. This known difference between groups should be reflected in the summarized hip and knee angular accelerations in case of a good discriminative validity.

Therefore, the study’s aim is to assess the discriminative validity of summarized hip and knee angular accelerations during a standardized training drill. Discriminative validity will be assessed in two ways. First, summarized hip and knee angular accelerations, and Playerload of national and regional soccer players will be compared over the full training drill and for parts that involve high-intensity efforts, passing and jumping header activities. Based on previous observations, it is hypothesised that national players would demonstrate higher training load over the full training drill and specifically for parts that involve high intensity running, passing or jumping header activities (Dellal et al. 2011). Second, discriminative validity will be assessed through examination of relationships between summarized hip and knee angular accelerations and conventional load indicators derived from a local positioning measurement system, such as high-intensity running distance or Playerload. Based on previous results, it is expected that summarized hip and knee angular accelerations will be unrelated to conventional indicators across the full training drill (Bastiaansen et al. 2022).

## Methods

### Participants

In this study, 12 national (Age: 22.2 ± 2.1 years, Weight 77.3 ± 7.3 kg, Length 1.83 ± 0.04 m, Training: 5.71 ± 5.4 hours/week) and 16 regional (Age: 24.1 ± 4.7 years, Weight 78.3 ± 8.6 kg, Length 1.87 ± 0.07 m, Training: 0.94 ± 1.1 hours/week) male soccer players participated. Categorization of players was based on previously described definitions (McKay et al. 2022). The national group consisted of highly trained (*n* = 4) and trained soccer players (*n* = 8), whereas the regional group were all recreationally active soccer players (*n* = 16). All participants were at least 16 years old, had more than one year soccer experience, and trained minimal once a week. None of the participants had any injuries during the study. Before participating, the study protocol was explained to the participants and informed consent was obtained. The study received approval from the local ethics committee of the Center for Human Movement Sciences (research register number: 202000503).

## Equipment

Five inertial measurement units (MPU-9150, Invensense, San Jose, California, USA) were placed at the lower back, both thighs and both shanks to capture lower extremity kinematics (Wilmes et al. 2020; Bastiaansen et al. 2020). One additional sensor was put within the socket between the shoulder blades (*i.e*. Local Position Measurement vest) to collect Playerload data, and one sensor was placed on a mechanical time gate to time-synchronize the data with LPM as outlined in procedures previously (Wilmes et al. 2022). Each sensor recorded 3D linear acceleration, 3D angular velocity, and 3D earth magnetic field strength at a sample rate of 500 Hz. The data was saved on a SD card embedded in a casing with a battery and the inertial sensor (size; 35 × 25x15 mm, weight; 11.0 g). Before measurements, the inertial sensors were time-synchronized following previously established procedures (de Ruiter and van Dieën 2019).

**Figure 1.**
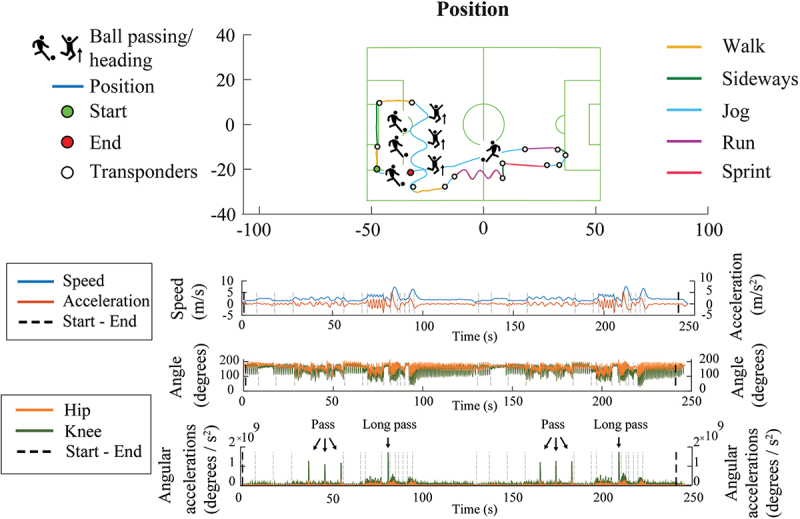
Illustrative example of standardized soccer training drill. Each colour represents a different activity.

An Inmotio Local Position Measurement system (Inmotio, Zeist, the Netherlands) was used to obtain player positional data on the pitch (Frencken et al. 2010; Stevens et al. 2014). The participants wore a vest with a transponder positioned between their shoulder blades including two antennas that transmitted a signal to ten base stations located around an artificial-turf soccer field. Two transponders were placed on the soccer field, to determine the start and end point of the training drill. Thirteen additional transponders were strategically placed to enable analysis of individual sections of the training drill (Figure 1).

### Procedures

This cross-sectional cohort study was conducted between February 2021 and May 2022. Players went through a 10-minute warm-up regimen that included jumping, kicking, and runs at submaximal and maximal pace. This was followed by a calibration process, which involved a 5-second stationary calibration where they stood upright, followed by a dynamic calibration which involved alternated raising left and right legs and bending forward (Wilmes et al. 2020; Bastiaansen et al. 2020). Then, participants performed three synchronisation runs to synchronise measurement systems (Wilmes et al. 2022). Participants ran through a mechanical time gate with inertial sensor, and two active LPM transponders that were placed on the ground. This caused a peak in both measurement systems. The average time difference was used to time synchronise both systems.

The experimental task was based on a soccer training drill that replicates the demands of the game (Kelly et al. 2013). Participants were instructed to perform seven different locomotor activities, combined with soccer specific activities in a fixed order (Figure 1). Activities consisted of walking forward or backward, jogging forward or backward, sideways movement, running, and sprinting. In addition, participants were instructed to pass the ball to a target, to head the ball, or to make a long pass towards the goal (Table 1). A ball was placed at the end of the drill, so that participants could dribble with the ball back to the start. The participants were verbally supported by the researcher during the drill to ensure the protocol was well executed. Then, 4 × 4–minute exercise bouts of the training drill were performed. The first 4 minutes were used as familiarization, which were followed by three experimental trials. Between trials, participants had 3 minutes of active rest where they dribbled with the ball.

**Table 1.** Verbal cues and instructions provided to participants.

Verbal cue	Instruction provided
Walk forward/backward	Walk at low intensity in forward/backward direction.
Jog forward/backward	Jog at low intensity in forward/backward direction.
Sideways	Move into a sideways direction at jog intensity.
Run	Run at 80% of maximal intensity in forward direction.
Sprint	Sprint at maximal intensity in forward direction.
Pass	The ball is placed on the ground. Pass the ball forwards to a specific target on ipsilateral side.
Long pass	The ball is placed on the ground. Perform a long pass in forward direction towards the goal, that serves as a target.
Jumping header	The researcher acts as ball server. Make a jumping movement to head the ball back towards the researcher, followed by a landing movement and continue the course.
Dribble	A ball is placed at the end of the drill. Dribble back with the ball at low intensity.

### Data processing

#### Inertial sensor setup

Lower extremity kinematics were obtained using gyroscope data, using a second order low-pass Butterworth filter with a cut-off frequency of 8 Hz (Bastiaansen et al. 2022). The orientation of the sensors relative to the global earth frame was determined using a gradient descent algorithm (Madgwick et al. 2011). Joint angles and angular velocities were then obtained by expressing the difference in sensor orientation between distal and proximal body segments following procedures described in Wilmes et. al (Wilmes et al. 2020). Joint angular accelerations were obtained by differentiation of joint angular velocities.

Lower extremity training load was based on angular accelerations across the hip and knee joint of the preferred kicking leg. The outcomes in this study are the cumulative sum of squared magnitude joint accelerations (α_hip, knee_). The magnitude was squared to give high-intensity movements more weight to the estimate, since these movements are more demanding for musculoskeletal tissues. The squared magnitudes were then divided by an arbitrary scale factor to improve readability, and thus expressed in arbitrary units (equation 1);

(1) $$\mathrm{Summarized}\;\mathrm{hip}\;\mathrm{or}\;\mathrm{knee}\;\mathrm{angular}\;\mathrm{accelerations}={\frac{\left|\alpha_{\mathrm{hip},\mathrm{knee}}\right|}{10^{8}}}^{2}$$

Where α_hip,knee_ denotes the measured 3D angular acceleration magnitude of the hip or knee joint at each time-point.

Playerload was calculated from 3D linear accelerations of the trunk inertial sensor, which were filtered by a 4th order low-pass Butterworth filter with a 10 Hz cut-off frequency and expressed by equation 2 (Boyd et al. 2011).

(2) $$\mathrm{Playerload}=\frac{\sqrt{{\left(a_{x}-a_{x-1}\right)}^{2}+{\left(a_{y}-a_{y-1}\right)}^{2}+{\left(a_{z}-a_{z-1}\right)}^{2}}}{100}$$

## LPM system

Inmotio software (version v6.2.0.383, Inmotio, Zeist, The Netherlands) was used to extract data at 50 Hz with a Weighted Gaussian average filter set at 85%. Training load indicators used for further analyses were total distance covered, high-intensity running distance (15–19.8 km/h), very high intensity running distance (19.8–25.1 km/h), and sprinting distance (>25.1 km/h). In addition, high-intensity acceleration (>2.78 m/s^2^) and high-intensity deceleration distance (<2.78 m/s^2^) were calculated (Saeterbakken et al. 2019).

### Statistical procedures

The average cumulative training load of the first, second and third 4-minute experimental trial were calculated and included for statistical analysis. The average of the three trials was then used to assess differences in training load between national and regional soccer players over the full training drill with a MANOVA test. This was followed by independent t-tests if MANOVA was significant. To compare training load indicators in specific parts of the training drill, cumulative load indicators were normalised to distance. To assess between-group differences per intensity, activities were categorized as walk, jog, sideways, high-intensity run, or sprint. The sub-parts within the training drill at jog intensity were used to assess between-group differences per activity. The activities were categorized as jog without ball, jog with jumping header and passing, and dribble with the ball. Repeated measures ANOVA tests were then used to assess influence for interaction effects between playing level and intensity or additional soccer-specific activities. The Greenhouse-Geisser correction was used when assumption of sphericity was violated. Partial eta squared and 95% confidence intervals were reported to interpret differences. Post hoc independent t-tests with Benjamini-Hochberg correction were used in case of significance to determine between group differences per soccer activity. These statistical procedures were also followed for Playerload, allowing for comparison between training load indicators.

Second, relationships between the summarized hip and knee angular accelerations and conventional load indicators were assessed with Pearson correlation coefficient, according to Cohen’s recommendations (Hopkins et al. 2009). Data were checked for normality beforehand with Shapiro test, and Q-Q plots. R Studio (Version 3.0.3) was used for statistical analysis and significance was accepted when *p* < .05.

## Results

Anthropometric characteristics did not differ between groups, but national players significantly trained more hours per week than regional soccer players (Mean difference: 4.77 hours, 95% CI: 1.3 to 8.2 hours, ES = 1.31, *p* < 0.001).

Descriptive statistics of load indicators for the full training drill are presented in Table 2. The MANOVA test revealed a significant effect of playing level (*p* = 0.001, η_p_^2^ = 0.72). Compared to regional soccer players, national players showed higher training load values for all indicators, except total distance and high intensity running distance (Table 2).

**Table 2.** Summarized hip and knee angular accelerations and whole-body training load indicators (means ± SD) for national and regional soccer players over the total standardised training drill. Mean difference, 95% confidence interval and Cohen’s effect sizes are presented, with significant (*p* < 0.05 between-group differences displayed in bold.

Indicator	National			Regional			Mean difference	95% CI	P	Effect Size (Cohen’s D)		
*IMU*												
**Hip (A.U.)**		**491 ± 95.5**			**428 ± 64.5**		**62.7**	**0.2–125.2**	**0.049**	**0.77**	**Moderate**	
**Knee (A.U.)**		**999 ± 151**			**862 ± 102**		**137.1**	**38.1–236.1**	**0.008**	**1.06**	**Large**	
**Playerload (A.U.)**		**47.7 ± 6.3**			**41.7 ± 5.5**		**6.0**	**1.4–10.6**	**0.01**	**1.02**	**Large**	
*LPM*												
Total distance (m)		569 ± 41.1			548 ± 36.8		20.5	−9.7–50.8	0.17	0.53	Moderate	
High Intensity Running Distance (15–20 km/h, m)		35.8 ± 9.7			35.5 ± 8.2		0.32	−6.6–7.2	0.92	0.04	Negligible	
**Very High Intensity Running Distance** **(20–25 km/h, m)**		**39.3 ± 4**			**21.7 ± 13**		**17.5**	**9.2–25.8**	**<0.001**	**1.67**	**Large**	
**Sprint distance (m)**		**9.5 ± 9.2**			**1.01 ± 3.3**		**8.5**	**3.2–13.7**	**0.002**	**1.22**	**Large**	
**Acceleration distance (m)**		**8 ± 1.3**			**4.98 ± 1.8**		**3.0**	**1.8–4.3**	**<0.001**	**1.90**	**Large**	
**Deceleration distance (m)**		**4.8 ± 3.7**			**1.73 ± 1.9**		**3.1**	**0.8–5.3**	**0.008**	**1.05**	**Large**	

The cumulative sum of normalised hip and knee angular accelerations is displayed in Table 3. Both hip and knee angular accelerations were sensitive for intensity (Hip: *p* < 0.001, η_p_^2^ = 0.49; Knee: *p* < 0.001, η_p_^2^ = 0.39) and soccer-specific activities (Hip: *p* < 0.001, η_p_^2^ = 0.38; Knee: *p* < 0.001, η_p_^2^ = 0.59). Post hoc analysis revealed that normalised hip and knee angular accelerations increased in all conditions when compared to walking. Moreover, normalised hip and knee angular accelerations increased with additional passing or jumping header and long pass activities.

**Table 3.** Distance normalised hip and knee angular accelerations, and Playerload values (means ± SD) of national and regional soccer players. Mean difference, 95% confidence interval and Cohen’s effect sizes are presented, with significant (*p* < 0.05) between-group differences displayed in bold.

*Activity*	National			Regional			Mean difference	95% CI	P	Effect Size (Cohen’s d)	
*Hip (A.U./m)*											
**Walk**		**0.37 ± 0.1**			**0.44 ± 0.1**		**− 0.07**	**-0.4–0.4**	**0.02**	**−0.57**	**Moderate**
Sideways		**0.83 ± 0.3**			**0.85 ± 0.3**		− 0.02	−0.2–0.1	0.75	−0.07	Negligible
**Jog**		**0.85 ± 0.2**			**0.75 ± 0.2**		**0.1**	**0–0.2**	**0.02**	**0.43**	**Small**
**Run**		**1.17 ± 0.3**			**0.93 ± 0.2**		**0.2**	**0.1–0.4**	**0.005**	**0.98**	**Large**
**Sprint**		**1.35 ± 0.3**			**1.08 ± 0.3**		**0.3**	**0.1–0.5**	**0.02**	**1.01**	**Large**
*Knee (A.U./m)*											
Walk		0.93 ± 0.2			1.02 ± 0.3		−0.09	−0.2–0	0.09	−0.41	Small
Sideways		**1.63 ± 0.9**			**1.67 ± 0.8**		−0.04	−0.4–0.3	0.81	−0.05	Negligible
Jog		1.66 ± 0.4			1.57 ± 0.3		0.08	0–0.2	0.22	0.23	Small
**Run**		**2.40 ± 0.4**			**1.79 ± 0.3**		**0.61**	**0.4–0.8**	**<0.001**	**1.52**	**Large**
**Sprint**		**2.57 ± 0.4**			**2.01 ± 0.4**		**0.56**	**0.3–0.8**	**<0.001**	**1.57**	**Large**
*Playerload (A.U./m)*											
Walk		0.047 ± 0.01			0.052 ± 0.03		−0.005	−0.02–0.05	0.37	−0.22	Small
Sideways		0.087 ± 0.01			0.086 ± 0.02		0.0006	−0.006–0007	0.85	0.04	Negligible
Jog		0.095 ± 0.01			0.089 ± 0.02		0.006	0.0005–0.01	0.11	0.38	Small
Run		0.080 ± 0.02			0.073 ± 0.02		0.007	−0.002–0.02	0.21	0.43	Small
Sprint		0.081 ± 0.01			0.070 ±0.02		0.01	−0.0001–0.02	0.11	0.79	Moderate
*Hip (A.U./m)*											
Jog no ball		**0.87 ± 0.2**			**0.75 ± 0.2**		0.12	0–0.2	0.06	0.56	Moderate
Dribble		**0.65 ± 0.2**			**0.59 ± 0.2**		0.05	−0.1–0.2	0.56	0.32	Small
Sideways + long pass		1.16 ± 0.3			1.12 ± 0.3		0.04	−0.2–0.3	0.68	0.16	Negligible
Jog + pass + jumping header		1 ± 0.2			0.94 ± 0.1		0.06	−0.1–0.2	0.56	0.39	Small
*Knee (A.U./m)*											
Jog no ball		**1.64 ± 0.4**			**1.54 ± 0.3**		0.09	−0.1–0.3	0.58	0.27	Small
Dribble		**1.43 ± 0.2**			**1.38 ± 0.3**		0.05	−0.1–0.2	0.58	0.21	Small
Sideways + long pass		2.73 ± 0.4			2.57 ± 0.5		0.16	−0.2–0.5	0.58	0.35	Small
Jog + pass + jumping header		1.94 ± 0.3			1.86 ± 0.3		0.07	−0.1–0.3	0.58	0.29	Small
*Playerload (A.U./m)*											
Jog no ball		0.097 ± 0.01			0.093 ± 0.02		0.12	−0.003–0.01	0.63	0.25	Small
**Dribble**		**0.093 ± 0.01**			**0.070 ± 0.02**		0.05	0.005–0.03	**0.02**	**1**	**Large**
Sideways + long pass		0.101 ± 0.01			0.099 ± 0.01		0.04	−0.008–0.01	0.71	0.14	Negligible
Jog + pass + jumping header		0.097 ± 0.01			0.094 ± 0.01		0.06	−0.008–0.01	0.71	0.17	Negligible

**Figure 2.**
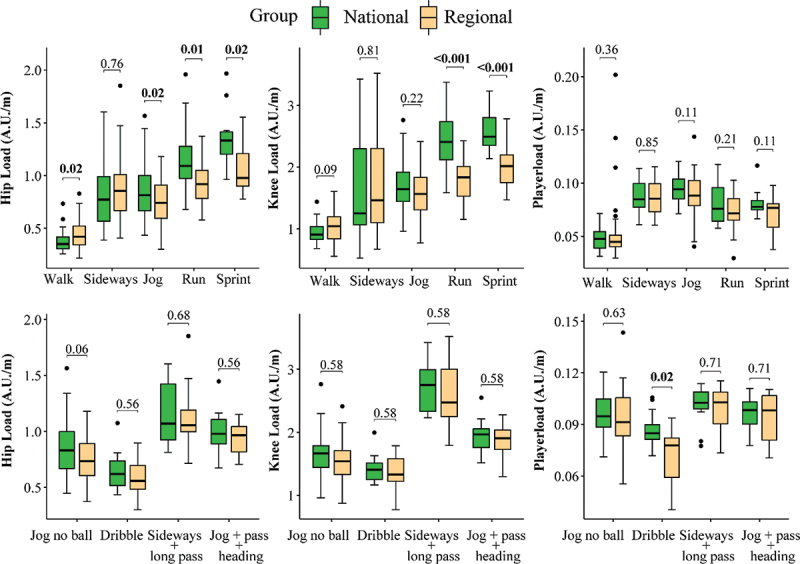
Summarized hip and knee angular accelerations, and Playerload values normalised to distance for each intensity and activity.

Significant interaction effects between intensity and playing level were observed in both normalised hip angular accelerations (*p* < 0.001, η_p_^2^ = 0.08) and knee angular accelerations (*p* < 0.001, η_p_^2^ = 0.08) (Figure 2). National players had higher hip angular accelerations compared to regional players during jogging, high intensity running, and sprinting (Table 3). Knee angular accelerations were higher for national players during high intensity running (Table 3). However, no significant interaction effects were observed for tasks that involved additional kicking and jumping header or long pass activities (Hip: *p* = 0.09, η_p_^2^ = 0.09; Knee: *p* = 0.48, η_p_^2^ = 0.02).

The normalised Playerload values are presented in Table 3. There were significant effects of intensity (*p* < 0.001, η_p_^2^ = 0.45). All intensity parts differed, except for sideways and jogging, and high intensity running and sprinting. No significant group-intensity interaction effects were observed (*p* = 0.06, η_p_^2^ = 0.03, Figure 2). Significant task effects were observed (*p* < 0.001, η_p_^2^ = 0.22). However, Playerload was unable to differentiate parts that involved jogging without ball, sideways + long pass, and jumping header + passing activities. A significant task-group interaction effect (*p* = 0.02, η_p_^2^ = 0.07) was observed. National players showed higher Playerload values in the part that involved a dribble with the ball.

Relationships between the training load indicators are displayed in Table 4. The summarized hip and knee angular accelerations were not significantly related to all other load indicators.

**Table 4.** Correlations of training load indicators.

	Hip (A.U.)^a^	Knee (A.U.)^a^	Playerload (A.U.)	Total distance (m)	Jog distance (m)	HIRD (m)	VHIRD (m)	Sprint distance (m)
Hip (A.U.)^a^	1							
Knee (A.U.)^a^	0.67*	1						
Playerload (A.U.)	0.18	0.53	1					
Total distance (m)	0.27	0.47	0.48	1				
Jog distance (m)	0.25	0.36	0.39	0.92***	1			
HIRD (m)	0	0.28	0.22	0.56	0.49	1		
VHIRD (m)	0.4	0.46	0.53	0.55	0.33	−0.09*	1	
Sprint distance (m)	0.05	0.36	0.49	0.5	0.31	0.13	0.44	1

*p<0.05; ** *p*<0.01; *** *p*<0.001; ^a^ summarized angular accelerations; HIRD = high intensity running distance (15–19.8 km/h); VHIRD = very high intensity running distance (19.8–25 km/h).

## Discussion

This study aimed to assess the discriminative validity of summarized angular acceleration-based training load indicators, during a standardized training drill. In line with the hypotheses, national players had higher summarized hip and knee angular accelerations than regional players over the full standardized training drill. Significant interaction effects were observed for high-intensity running and sprint activities, with higher summarized hip and knee angular accelerations observed for national players. However, summarized hip and knee angular accelerations showed no interaction effects for tasks that involved passing or jumping header, which contradicts the expectations. Moreover, the insignificant relationships between the summarized hip and knee angular accelerations and the conventional indicators demonstrate they provide distinct information, which was in line with the expectations.

Over the full training drill, national players demonstrated greater summarized hip and knee angular accelerations which was in line with the expectations (Dellal et al. 2011). Furthermore, significant interaction effects were observed during high-intensity running and sprint activities. These results contradict previous observations where no differences for summarized hip and knee angular accelerations were observed during sprinting (Bastiaansen et al. 2023). The main difference between studies is that participants previously performed sprint tasks in isolation and had sufficient rest to reduce fatigue effects. The training drill in this study had a duration of approximately 30 minutes with active resting periods. Compared to the familiarization trial, the national players showed less reductions in maximal (−0.9 km/h vs. −1.3 km/h) and average speed (+0.4 km/h vs. −0.1 km/h). Therefore, fatigue has probably played a more dominant role in this study. Indeed, repeated sprint ability is considered a key performance factor in soccer (Rampinini et al. 2009; Dellal et al. 2011; Saeterbakken et al. 2019). The moderate to large differences in high-intensity running distance, while the total distance remained similar between groups substantiate these observations. Therefore, superior repeated sprint abilities of national players explain the difference in summarized hip and knee angular accelerations over the full protocol, and during running and sprint activities.

No significant interaction effects were visible for additional soccer specific activities, although the summarized hip and knee angular accelerations increased when players performed additional passing, jumping header, or long-passing activities compared to jogging. The increased summarized hip and knee angular accelerations are in line with previous observations in soccer players performing only running, running with additional kicking, and running with additional jumping (Wilmes et al. 2022). Players showed an increase in summarized hip and knee angular accelerations when kicking or jumping were included in the task. This might be the result of joint accelerations to transfer energy into the ball during passing activities (Naito et al. 2010), or moving the body upwards against gravity and to absorb the impact of landing during jump activities (Lees et al. 2004). However, the summarized hip and knee angular accelerations remained similar between groups during jumping header and passing activities. This contradicts previous observations where higher knee angular accelerations were observed for national players in a maximal instep kicking task without accuracy demands (Bastiaansen et al. 2023). Because in the present study the emphasis was more on accuracy instead of on maximal physical performance the contrast in joint angular accelerations between groups probably disappeared (van den Tillaar and Fuglstad 2017). Additionally, national players could have performed the activities more efficiently, given their superior technical abilities in performing soccer-specific tasks (Klingner et al. 2021; Boyne et al. 2021). Both factors might explain why similar load was observed between groups during soccer-specific passing and jumping header activities in the training drill.

The summarized hip and knee angular accelerations were unrelated to the conventional load indicators, which is in line with previous observations (Bastiaansen et al. 2022). The insignificant relationships between measurement systems indicate that both the inertial sensor setup and local position registration provide unique information on training load and can therefore be used in conjunction. Furthermore, Playerload was unable to discriminate players at high intensity activities, where the summarized hip and knee angular accelerations do. This result is probably because the summarized hip and knee angular accelerations were squared, giving more weight to peaks in angular accelerations in the outcome. This can be seen as a strength because high-intensity movements might be an important factor for injury incidence. Contrary to the summarized hip and knee angular accelerations, Playerload was not able to discriminate parts that involved jogging, long passing, and passing and jumping header activities. Since the summarized hip and knee angular accelerations are specific for the lower extremities, these may reflect lower extremity training loads of soccer players better when compared to whole-body load indicators (Wilmes et al. 2022; Bastiaansen et al. 2022). The whole-body indicators derived from these systems may better reflect the physiological load soccer players endure. As such, multiple training load indicators might be used to provide a holistic view on physiological and biomechanical loads during training and match play. This can help to better balance load and recovery, prevent overload, and reduce injury risk, although this remains to be established in future studies.

Differences in training load between national and regional soccer players and between measurement systems were confirmed, supporting the validity and use of summarized hip and knee angular accelerations as lower extremity training load indicators in the field. The standardised training drill enabled to test conditions under ecologically valid circumstances that better replicate the game demands compared to previous studies. Trainers, coaches, and professionals can use this information to evaluate and adjust training to achieve optimal player performance. However, it is currently not feasible to use the inertial sensor setup daily because the method is labour intensive. To overcome this problem, the inertial sensor setup can be integrated in shorts or tights (Steijlen et al. 2021).

A limitation of this study is that assessments were based on training load indicators only. Participants were instructed to pass the ball accurately towards a specific direction, but the outcome whether passing was successful or not was not considered for the analysis which might have influenced results. Second, it remains to be established in future studies how the summarized hip and knee angular accelerations could help performance optimization strategies, since this relationship is not examined yet. Likewise, exploring the association with injury risk is needed for a better understanding of its implications in daily practice. Moreover, this study followed a cross-sectional design and group-based analysis. A next step could be to monitor training load longitudinally, such as the ‘Return to Sport’ process.

## Conclusion

The discriminative validity of summarized hip and knee angular accelerations for lower extremity training load quantification during a standardized training drill is confirmed. Over the full training drill, national players had higher hip and knee angular accelerations compared to regional players. Higher hip and knee angular accelerations were observed during high-intensity running and sprint activities, but not during passing, jumping header or long passing activities. However, Playerload had less discriminative validity than the summarized hip and knee angular accelerations. Furthermore, the summarized hip and knee angular accelerations were unrelated to conventional methods which confirmed the discriminative validity between measurement systems. These results further support the validity of summarized hip and knee angular accelerations as external training load indicators to evaluate soccer training and match play.
